# Multilocular Unicystic Ameloblastoma of Mandible

**DOI:** 10.1155/2013/835892

**Published:** 2013-09-10

**Authors:** Manas Bajpai, Deshant Agarwal, Anindya Bhalla, Malay Kumar, Rakesh Garg, Manish Kumar

**Affiliations:** ^1^Department of Oral and Maxillofacial Pathology, NIMS Dental College, Jaipur, India; ^2^Department of Public Health Dentistry, NIMS Dental College, Jaipur, India; ^3^Department of Oral and Maxillofacial Pathology, Ahmedabad Dental College, Ahmedabad, India; ^4^Department of Periodontics, NIMS Dental College, Jaipur, India; ^5^Department of Prosthodontics Crown and Bridges, NIMS Dental College, Jaipur, India

## Abstract

*Introduction*. We report a rare case of unicystic ameloblastoma (UA) of mandible which showed multilocular radiolucency on the left side of mandible on radiographic examination which is very unusual, and the majority of the cases of UAs till date has been reported of unilocular radiolucency. On histopathological examination, an odontogenic cystic lining that proliferates that intraluminally resembling ameloblastomatous epithelium was observed, leading to a definitive diagnosis of unicystic ameloblastoma. *Case Presentation*. A 42-year-old male patient presented with a swelling on the left side of the mandible extending from 33 to 36. Radiographically, it showed a multilocular radiolucent lesion resembling odontogenic cyst; however, the final diagnosis was made on histopathological ground with the inclusion of radiological and clinical features. *Conclusion*. It can be concluded that at present, histopathologic examination is the most sensitive tool for differentiating between odontogenic cysts and UAs. However, both clinical and radiologic findings share equal contribution to the final diagnosis.

## 1. Introduction

Ameloblastomas are benign tumors whose importance lies in its potential to grow into enormous size with resulting bone deformity. They are typically classified as unicystic, multicystic, peripheral, and malignant subtypes [1]. A solid or multicystic ameloblastoma is a benign epithelial tumor of odontogenic origin showing a strong tendency to recurrence and local aggression. Solid/multicystic, peripheral, desmoplastic, or unicystic ameloblastomas are other subtypes of ameloblastoma [2, 3]. 

 Unicystic ameloblastoma (UA) represents an ameloblastoma variant, presenting as a cyst [3]. 

In 1977, Robinson and Martinez first used the term “unicystic ameloblastoma” (UA) for such lesions [4], but it was adopted in the second edition of the international histologic classification of odontogenic tumors by the WHO in 1992 [5]. The other name as recognised by WHO is “cystogenic ameloblastoma” [3]. 

Five to 15% of all ameloblastomas are of the unicystic type. Cases associated with an unerupted tooth show a mean age of 16 years as opposed to 35 years in the absence of an unerupted tooth. The mean age is significantly lower than that for solid/multicystic ameloblastoma. There is no gender predilection [3]. Unicystic ameloblastoma (UA) is a prognostically distinct entity. It has a recurrence rate of 6.7–35.7%, and the average interval for recurrence is approximately 7 years.

Six radiographic patterns have been identified for UA, ranging from well-defined unilocular to multilocular ones. When the radiographic appearance is divided into the two main patterns, unilocular and multilocular, there is a clear predominance of a unilocular configuration in all studies of UA where this feature has been evaluated, especially in cases associated with impacted teeth [6].

## 2. Case Presentation

**Figure 1:**
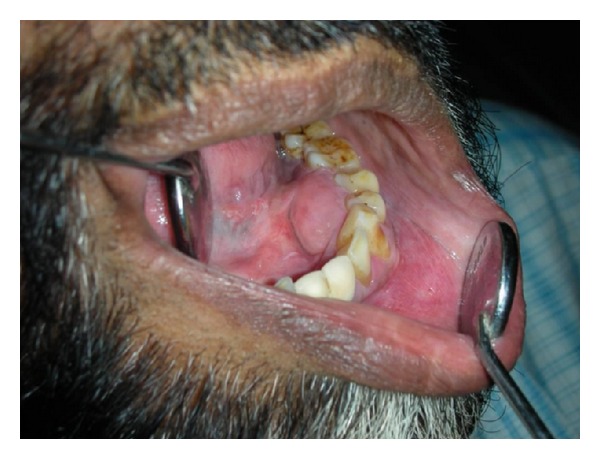
Swelling on lingual surface on the left side of the mandible extending from 33 to 36.

**Figure 2:**
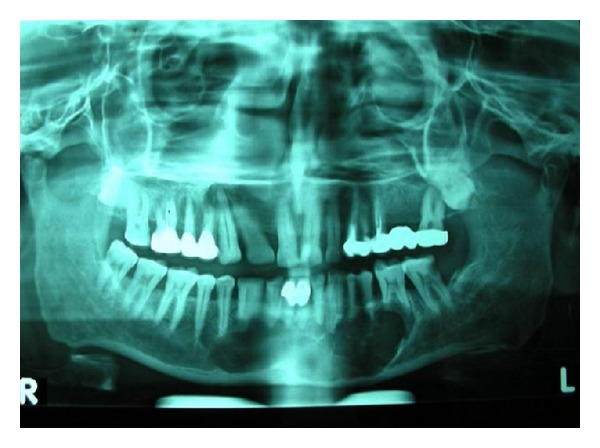
OPG of the patient reveals a large multilocular radiolucency.

**Figure 3:**
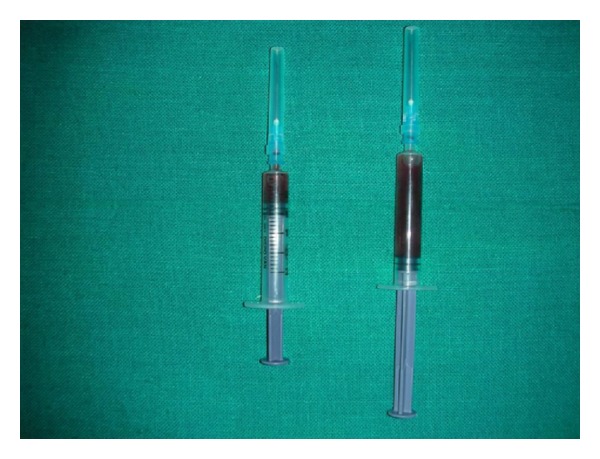
Serosanguinous aspirate with blood.

A 42-year-old male patient presented with the chief complains of swelling on the left side of the mandible since 1 month. On intraoral examination, solitary, oval swelling measuring 4 × 2 cm in size seen on the left side of the mandible extending from 33 to 36 was noticed (Figure 1). The swelling was hard in consistency with smooth surface, diffuse margins and was nontender on palpation. The buccal and lingual cortices were thinned out. Pain associated with the swelling was insidious in origin and dull, nonradiating, and intermittent in nature. A provisional diagnosis of odontogenic keratocyst was given. Panoramic radiograph revealed a huge osteodestructive lesion with well-defined multilocular radiolucency with thin corticated borders in the left side of the mandible extending from 36 to 45 (Figure 2). On fine needle aspiration, a serosanguinous aspirate was obtained which revealed acute inflammatory cells (consisting mainly of polymorphonuclear neutrophils) and cystic macrophages in an eosinophilic background on cytopathologic examination (Figure 3). Incisional biopsy was carried out to obtain a definitive diagnosis.

**Figure 4:**
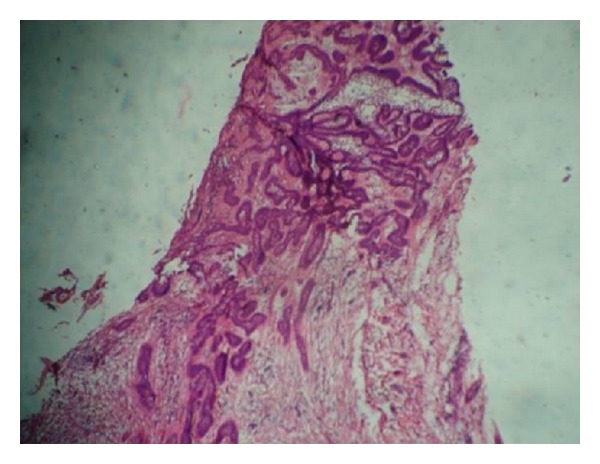
Cystic lumen lined by nonkeratinized stratified squamous epithelium showing prominent basal cells (H and E) 10x.

**Figure 5:**
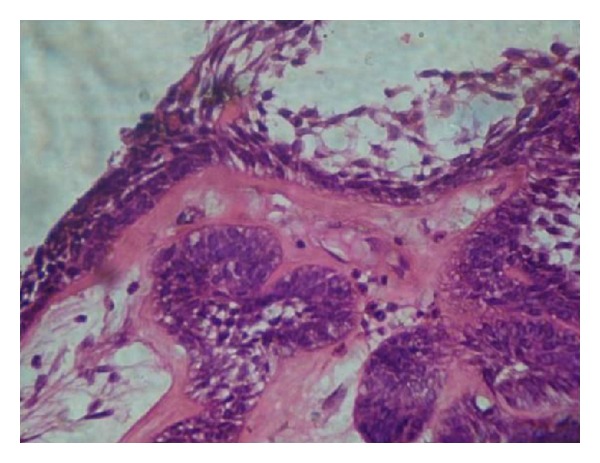
Suprabasal layer resembles stellate reticulum proliferating intraluminally with few vacuolated cells. (H and E) 40x.

**Figure 6:**
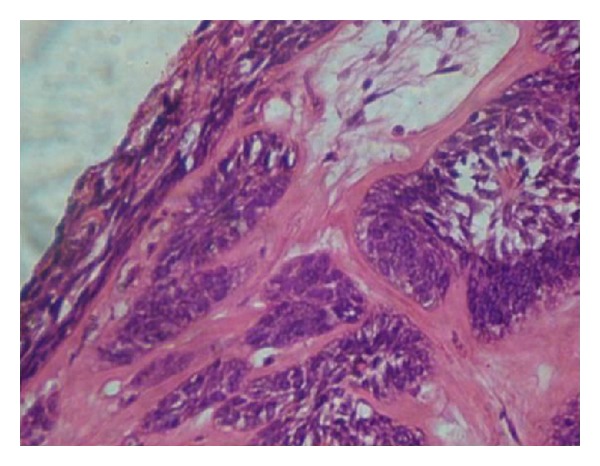
Squamous metaplasia of cystic lining and hyalinization of underlying connective tissue (H and E) 40x.

Histopathological examination demonstrated cystic lumen lined by nonkeratinized stratified squamous epithelium showing prominent basal cells with hyperchromatic nuclei which is polarized (Figure 4). The suprabasal layer resembles stellate reticulm like cells which are proliferating intraluminally with few vacuolated cells (Figure 5). The cystic lining shows squamous metaplasia of varying thickness at many places. The underlying connective tissue capsule shows inductive hyalinized eosinophilic band just beneath the epithelium (Figure 6). Thus, the histopathological findings confirmed the diagnosis of unicystic ameloblastoma (Table 1).

## 3. Discussion

The term unicystic ameloblastoma has been described as an ameloblastoma developing within the lining, lumen, or wall of a cyst as well as an invasive ameloblastoma that has a single cystic space rather than multicystic spaces [8]. Unicystic ameloblastoma, a variant of ameloblastoma, was first described by Robinson and Martinez [4]. This terminology may also represent an odontogenic cyst in which there has been ameloblastic transformation of the epithelial lining [5].

Various contradictory theories about the development of UAs have been proposed (Table 1). While some authors suggest that UAs develop by cystic degeneration of solid ameloblastomas, there are certain indications that UAs may develop by mural and/or luminal ameloblastomatous change in a preexisting cyst [2]. Robert and Diane have proposed three pathogenic mechanisms for the evolution of unicystic ameloblastoma: reduced enamel epithelium, from dentigerous cyst and due to cystic degeneration of solid ameloblastoma [9]. 

Some cases are asymptomatic, sometimes presenting as a swelling of the posterior mandible. More than 90% of cases involve the mandible, usually the posterior region. Up to 80% are associated with an unerupted mandibular third molar. The lesion presents radiographically as a well-corticated unilocular pattern, often pericoronal radiolucency [3].

The unilocular pattern is more common in the unicystic variant than the multilocular one, especially so in cases associated with tooth impaction [7].

Unicystic ameloblastoma may mimic other odontogenic cysts clinically and radiographically. Moreover, the histologic distinction between UAs and certain nonneoplastic odontogenic cysts can be difficult [10]. It appears to be more difficult to differentiate UAs in cases of dentigerous UAs (associated with an impacted tooth) than in cases of nondentigerous UAs (not associated with an impacted tooth). UAs that are not associated with an impacted tooth may mimic a residual cyst or a keratocystic odontogenic tumor (KCOT) [2]. Much confusion stems from the fact that a UA may appear not only as a unilocular but indeed also as a multilocular bone defect [7].

Since 1925, many had reported the development of ameloblastoma within the walls of odontogenic cysts, and the most commonly cited was the dentigerous cyst [4]. There have been many reports of ameloblastomas apparently arising from the epithelium of what initially was considered as an odontogenic cyst [7]. Hyperplastic epithelium may also resemble ameloblastomatous lining epithelium in radicular cyst and dentigerous cyst. However, since this type of feature was also associated with a dense inflammatory cell infiltrate where the stellate-reticulum like epithelium was a result of intercellular oedema arising from the presence of chronic inflammation in the area, it should be considered as not diagnostic of unicystic ameloblastoma [4]. In the present case, a lining epithelium resembling dentigerous cyst lining was evident in one specimen. However, a characteristic ameloblastomatous lining was evident on taking deeper sections of the other specimen that confirmed the diagnosis of unicystic ameloblastoma. This case, thus, highlights the role careful microscopic examination of all the sections in the biopsy specimen and of deeper and step sections in the diagnosis of unicystic ameloblastoma as also has been shown by Dunsche et al. [2]. In the past, other authors had suggested that in cases of small islands of ameloblastomatous epithelium within the cystic epithelium of a lesion, it might be necessary to examine the entire specimen to be sure of finding these islands [7, 11, 12].

In present case, the lesion presented as a localised swelling in the mandible and multilocular radiolucency on radiographic examination, which resembled a solid multicystic ameloblastoma or keratocystic odontogenic tumor (KCOT), and was difficult to diagnose clinically and radiographically. The final diagnosis was made on the basis of histopathological examination and also by the correlation of clinical and radiological features.

## 4. Conclusion

It can be concluded that at present, histologic examination is the most sensitive tool for differentiating between odontogenic cysts and UAs. However, both clinical and radiologic findings share equal contribution to the final diagnosis. This case also highlights the importance of careful examination of the entire specimen and the usefulness of deeper sections in diagnosis of unicystic ameloblastoma. Thus, it is of utmost importance to correlate the histopathologic findings with clinical and radiographic features to achieve at a correct definitive diagnosis as all such lesions may have prognostically different biologic behaviours and the final diagnosis may alter the therapeutic decision significantly.

## Figures and Tables

**Table 1:** Histopathological classification of unicystic ameloblastomas (UAs) [7].

Group	Interpretation
1	Luminal UA
1.2	Luminal and intraluminal UAs
1.2.3	Luminal, intraluminal, and intramural UAs
1.3	Luminal and intramural UAs
